# Willingness to Use Digital Health Tools in Patient Care Among Health Care Professionals and Students at a University Hospital in Saudi Arabia: Quantitative Cross-sectional Survey

**DOI:** 10.2196/18590

**Published:** 2021-02-19

**Authors:** Subash Thapa, Jesper Bo Nielsen, Abdullah M Aldahmash, Fatima R Qadri, Anja Leppin

**Affiliations:** 1 Research Unit of General Practice Department of Public Health University of Southern Denmark Odense Denmark; 2 Prince Naif Bin Abdulaziz Health Research Center King Saud University Riyadh Saudi Arabia; 3 College of Medicine, Alfarabi Colleges Riyadh Saudi Arabia; 4 Unit for Health Promotion Research, Department of Public Health University of Southern Denmark Esbjerg Denmark

**Keywords:** attitude, digital health, electronic medical record, health care professionals, health care students, Saudi Arabia, self-efficacy, telemedicine, willingness to use

## Abstract

**Background:**

The adoption rate of digital health in the health care sector is low in many countries. A facilitating factor for successful implementation and adoption of digital health is acceptance by current and future health care professionals.

**Objective:**

This study was conducted to identify factors associated with willingness to use digital health tools in patient care among health care professionals and students.

**Methods:**

This was a quantitative cross-sectional survey study conducted among health care professionals and students at a university hospital in Riyadh, Saudi Arabia. A nonprobability convenience sampling procedure was used to recruit participants. Data were collected using a self-completed e-questionnaire that was distributed by email. Chi-square tests, *t* tests, and logistic regression were used to analyze the data.

**Results:**

We found that 181 out of 218 health care professionals (83.0%; 75.6% [59/78] physicians; 87.1% [122/140] nurses) and 115 out of 154 students (74.7%; 80.0% [76/95] medical students and 66.1% [39/59] nursing students) were willing to use digital tools in patient care. Willingness to use digital tools was significantly associated with attitude (Adjusted Odds Ratios [AOR] 1.96; 95% CI 1.14-3.36) and self-efficacy (AOR 1.64; 95% CI 1.17-2.30) among health care professionals, and with current year of study (AOR 2.08; 95% CI 1.18-3.68) and self-efficacy (AOR 1.77; 95% CI 1.17-2.69) among students. No significant difference in willingness to use digital tools was found between physicians and nurses (*P*=.113), and between medical and nursing students (*P*=.079).

**Conclusions:**

The findings of this study should encourage policy makers and hospital managers to implement relevant eHealth interventions within routine health care systems in Saudi Arabia. For successful implementation, digital health education programs should be implemented simultaneously, so that current and future health care professionals are able to develop required positive attitudes as well as practical skills and competencies.

## Introduction

The potential of digital health to support health systems in health care delivery, health promotion, and disease prevention has been recognized in many countries [1,2]. In hospital settings, digital health tools (also referred to as “eHealth tools”), such as patient–physician portals, telemedicine, electronic medical records, smartphone and tablet apps, or remote monitoring devices, can reduce demand for (in-house) consultations, medical procedures, and unnecessary hospitalizations as well as improve postoperative monitoring of patients [3,4]. In particular, digital tools may support self-management and preventive behaviors in patients with chronic conditions, such as diabetes, hypertension, asthma, or cardiovascular diseases [1,5].

Consequently, the use of eHealth tools in patient care is on the rise globally, as digital health interventions are being implemented in many countries [1,2,5]. Implementation quality and effectiveness, however, seem to vary widely by type of eHealth intervention and setting [5]. Digital health interventions can be challenging to implement, not only because they are often inherently complex but also because they may meet with a variety of barriers. Some impediments are systemic, such as lack of financial resources, poor fit with existing information and delivery systems, or disruption of established modes of interaction between health care professionals and patients [6]. Others are individual-level challenges, such as insufficient skills and competencies of health care professionals or unfavorable beliefs and expectations, such as using eHealth tools may create misunderstandings and mistrust in the patient–provider relationship or might limit professional autonomy or increase administrative burden [7,8].

Numerous studies have examined health care professionals’ willingness to use eHealth tools but level and quality of evidence in this area remain insufficient. This may partly be a consequence of an often narrow scope of individual studies, due to an exclusive focus on specific tools, such as telemedicine [9-12] and electronic medical record [13,14], one professional group (often medical doctors) [8,14-16], or one medical specialty [17-20]. Another reason is that findings often are discrepant [8,9,12-14,18,19,21,22]. For instance, many studies in the European region found low adoption rates when national strategies for the introduction of electronic medical records were first implemented [13,15,23], but some saw improvements over time [14,24], while others did not [12,25].

Similarly, health care professionals’ willingness to use digital tools has often but not always been found to be related to sociodemographic characteristics, such as age and gender or professional attributes [12,19,21,22,26]. Menachemi and Brooks [26], for example, reported that willingness to use computers and electronic medical records was significantly higher among male health professionals and those with longer years of professional experience. More recent studies by Saleh et al [19] and Grassl et al [12] noted that willingness varied between professional groups, with physicians being significantly more willing to use computers and telemedicine compared to other health professionals. Other studies, however, did not find significant differences in terms of age, gender, or professional education when it came to willingness to use various types of eHealth tools [16,17].

By contrast, there is large-scale consensus that sociocognitive factors, including attitudes toward eHealth tools and perceived benefits/costs as well as perceived ease of use, are important factors when it comes to health care providers’ willingness to use eHealth tools [10,13,15,16,20,27,28]. In particular, health care professionals’ perceptions that use of eHealth tools leads to improved communication as well as increased access to care and level of satisfaction among their patients have been found to lead to or be associated with higher willingness for adoption [20]. Perceived loss of autonomy and privacy, doubts about data safety, and anxiety about use, by contrast, seem to contribute to a lack of willingness [13,27].

When it comes to differences in implementation of eHealth on the country level, a main explanation might of course also be found in the wide variety between health care systems, quality of care, and specific eHealth strategies chosen, so that experiences may not necessarily be comparable between countries [29]. In fact, the World Health Organization (WHO) recommends that each country should have its own strategy to engage health care professionals in adopting digital health as part of their individual journey toward universal health coverage and patient-centered care [30].

Countries in the Gulf region, and Saudi Arabia in particular, are on their way to systematically introduce digital health systems, not at least due to an increasing burden of chronic, life style–related diseases. As many as 1 in 3 adults in Saudi Arabia is either obese or diabetic, and from 2000 to 2017, the population prevalence rates of diabetes increased from 26.2% to 34.5% in men and 21.5% to 28.6% in women [31]. In line with this, the proportion of people with cardiovascular risk factors, such as low-density lipoprotein cholesterol, hypertension, and low levels of physical activity, has increased over the recent decade, as reported by a recently published study [32]. The resulting increased demand for efficient patient management, in combination with the need of rural populations to cover large geographical distances to reach hospitals/care facilities [33], has led to an increased interest in eHealth tools and systems. Because population adoption rates of mobile phones/apps and use of social media are very high in the Saudi Arabian society, one might expect integration of eHealth into health care to be comparatively easy. However, not much is known yet about local health care professionals’ readiness to adopt eHealth tools in clinical practice and the factors associated with level of motivation.

To our knowledge, only 2 studies have investigated perceptions of eHealth and willingness to make use of these services in health care professionals working in Saudi Arabia [34,35]. The first study conducted by Albarrak et al [34] exclusively targeted physicians and found medium levels of knowledge about telemedicine and largely positive views toward using telemedicine. However, in that study, factors associated with willingness to use telemedicine in patient care were not investigated. The second study, by El-Mahalli et al [35], targeted physicians as well as other subgroups of health care professionals and investigated both willingness to use and actual adoption of telemedicine. It was noted that although the majority of health care professionals were willing to use telemedicine, the actual rate of adoption was low. Further, willingness to use telemedicine was not found to be associated with age, gender, professional education, and years of professional experience, while actual use was significantly higher among consultant physicians having more than 20 years of professional experience compared to more junior physicians and nonphysicians.

It is unclear to which extent these previous findings on willingness to use telemedicine can be generalized to other types of eHealth tools and devices. Besides, knowledge about readiness to use eHealth tools among other groups of health care professionals than medical doctors is insufficient. We believe that it is particularly relevant to include nurses, because they also play a major role in patient care. For a sustainable implementation of digital health services, it is further important that not only current health care professionals but also medical and nursing students as the future generation of health care providers are targeted [11,36]. Moreover, to our knowledge no study has as yet investigated whether and to which extent sociocognitive factors, such as eHealth-related attitudes, perceived benefits/costs, and self-efficacy, might function as potential barriers or facilitators for the willingness to use eHealth tools among health care professionals in Saudi Arabia.

Therefore, in this study, we aimed to investigate willingness to use digital tools in patient care among medical and nursing professionals and students in a clinical setting in Saudi Arabia. Further, we aimed to examine the associations of such willingness with sociodemographic and professional characteristics, with attitudes toward digital health tools in terms of their importance for patients’ care, as well as with general perceived costs and benefits of using these tools and with self-efficacy.

## Methods

### Study Design and Approval

This was a quantitative cross-sectional survey study conducted among health care professionals and students from King Saud University Medical City Hospital (KSUMC). KSUMC is one of the biggest tertiary level, multifacility, public hospitals in Riyadh, Saudi Arabia. Ethical approval was received from the Institutional Review Board of KSU College of Medicine (ethical approval number 18/0657/IRB).

### Study Participants

Physicians and nurses at KSUMC were targeted if they were employed in any of the following departments: internal medicine, cardiology, otolaryngology, obstetrics and gynecology, ophthalmology, orthopedics, pediatrics, psychiatry, intensive care unit, and surgery. These departments were chosen based on the rationale that they would have the highest potential to profit from the use of digital health tools.

The health care students included medical and nursing students from the College of Medicine and the College of Nursing, King Saud University (KSU), respectively. All medical and nursing students from the second year onward, as well as interns enrolled in the program were considered eligible. First-year students were excluded, as they would not yet have had relevant experience and direct contact with patients.

### Sampling and Recruitment

The hospital departments were contacted and informed about the aims of the planned research and asked for permission to conduct the study. All contacted departments gave permission and subsequently forwarded an invitational email to all physicians and nurses. There were altogether 864 eligible health care professionals (547 physicians and 317 nurses), all of whom received the invitation email. To reach out to the students, the academic coordinators at both colleges sent an invitational email on behalf of the research team to students from the second year onward. Altogether, 2143 students (1599 medical and 544 nursing students) were eligible, and all of them received the invitation email.

The invitation included information about the study and a web address that linked to an informed consent form. Subsequent to filling out the informed consent form, an e-questionnaire was sent out to the participants in May 2019. A reminder email was sent to nonresponders every second week over the 2-month recruitment period.

A total of 3007 health care professionals and students were sent the invitation email, 662 of whom participated in filling in an e-questionnaire (response rate 22.02%).

### Measurement

#### Questionnaire

The questionnaire was developed and administered via Survey-XACT. The dependent variable was “willingness to use digital health tools in patient care” and was measured by 1 question: “If digital health tools and services were (now or in the future) adopted by your department, would you be in favor of such a change?” (responses: 0=no, 1=yes, 2=not sure). The response “not sure,” which was endorsed 59 times, was merged with the “no” response, so that the final response categories were “not willing to use or uncertain about use” versus “willing to use.” This was based on the rationale that in the given cultural context it is often considered impolite to explicitly say “no.” Therefore, an expression of uncertainty might instead be used as a more acceptable way of giving a negative answer. In a similar vein, the alternative option to just offer a dichotomous “yes–no” response format was rejected, because social desirability tendencies might have motivated skeptical respondents to answer with “yes” rather than saying “no.” This would have led to even more serious misclassification effects.

The independent variables included sociodemographic characteristics, ever having received a training for digital health use, prior use of digital tools at the departmental level, attitudes toward using digital health tools, perceived costs and benefits of digital health tools, and self-efficacy regarding personally using digital health.

#### Sociodemographic Characteristics

These included age, gender, educational background (nursing/medicine), and professional background (nurse/physician/student). Additionally, we included number of years of direct contact with patients for health care professionals and current year of study for students.

#### Ever Received Training About the Use of Digital Health

This variable was based on the question: “Have you ever received any organized training or extended instructions about the use of digital health tools, which include patient–physician portals/websites, patient health records, remote monitoring devices, mobile apps, telemedicine, webinars, online encyclopedia and online peer groups?” (no/yes).

#### Use of Digital Tools at the Departmental Level

Prior experience was operationalized with 1 question: “Has your department ever implemented any digital health tools?” (no/yes).

#### Sociocognitive Variables

For the assessment of the sociocognitive variables, 3 new multi-item instruments were developed. A core team of 3 researchers (ST, FQ, and AL) reviewed qualitative and quantitative studies on health professionals’ perceptions regarding use of eHealth in patient care to identify a preliminary list of items for the assessment of “attitudes toward using digital health tools,” “perceived benefits/costs of digital health tools,” and “self-efficacy.” This pool of items was discussed in terms of content validity and core items selected accordingly. Subsequently, the remaining items were checked and improved in terms of their clarity/comprehensibility by the extended research team over several rounds of revision.

Finally, a pilot study with face-to-face cognitive interviews was conducted, including 2 physicians, 2 nurses, 1 medical student, and 1 nursing student. Based on the findings of these interviews, some items were edited to improve clarity and understanding, and redundant items were deleted.

##### Attitudes Toward Using Digital Health Tools

We developed a 10-item instrument, which specifically reflected the perceived relevance/value of different functions of digital tools for active engagement of patients in their own treatment/care. Example items are “How important would it be that your patients can use remote monitoring devices (eg, glucometer, oximeter) to monitor their clinical condition by themselves?” or “How important would it be that your patients can see their medical test results and the record of treatments they have received in patient portals/website?” All items were presented with 5-point scales (1=not important at all to 5=absolutely important). Item responses were summed up, and a mean score was computed. Cronbach α for the total scale was .93.

##### Perceived Benefits/Costs of Digital Health Tools

Altogether, 20 items were used to assess expectations about potential positive (10 items) and negative consequences (10 items) of introducing digital health tools in clinical care for patients, professionals, and for the hospital. Example items are “If digital health tools were introduced into clinical care in hospitals, quality of care will be...”; “If digital health tools were introduced into clinical care in hospitals, quality of communication between health care professionals and patients will be....” All items were to be rated on 5-point Likert scales (1=much lower to 5=much higher). The subgroup of 10 items relating to the positive consequences of using digital tools was labeled as “perceived benefits,” while the 10 items relating to the potential psychological, financial, technological, and administrative burden of using digital tools were labeled as “perceived costs.” Item responses for perceived benefits and costs were summed up, and the means of the respective sum scales were used as final scores. Cronbach α was .89 for the perceived benefits scale and .81 for the perceived costs scale.

##### Self-Efficacy

We developed a 12-item instrument, which reflected the belief in one’s own ability to successfully perform various specific actions related to the use of digital tools in patient care. All items were presented with response scales from 0 to 6 (0=not at all confident to 6=100% confident). An example item is: “How confident are you that you are able to monitor the patients’ health data using mobile apps.” All items were summed up, and the mean of the total scale was used as final score. Cronbach α for the scale was .94.

Because most health care professionals in KSUMC are expatriates, the questionnaire was made available in English only.

### Data Analysis

Descriptive statistics were used to present all variables (percentages, means, and SDs). For bivariate analysis, independent samples *t* tests were conducted to compare the means for perceived benefits/costs of using digital tools in patient care between health care professionals and students. Further, chi-square tests and *t* tests were conducted separately for the samples of professionals and students to examine the bivariate associations of sociodemographic variables, ever having received eHealth training, experience of using digital tools at the departmental level, attitudes, perceived costs/benefits, and self-efficacy with the willingness to use digital tools in patient care. A *P-*value <.05 was considered statistically significant.

On the multivariable level, logistic regression analysis was used to identify individual factors associated with willingness to use digital health tools in patient care among health care professionals and students while adjusting for effects of potential other influencers. To maximize power, only factors associated with the dependent variable at a level of *P*<.10 in the respective bivariate analysis were carried forward to the multivariable logistic regression models. Adjusted Odds Ratios (AOR) and 95% CIs were calculated. All the data were analyzed using SPSS 24.0 for Windows (IBM).

## Results

### Overview

Among the group of nonresponders (n=290), students as compared to health care professionals (*P*<.001), male participants (*P*<.001), and younger ones (*P*<.001) were significantly less likely to complete the e-questionnaire (Multimedia Appendix 1). Questionnaires with missing values in the dependent variable, that is, willingness to use eHealth tools, and attitudinal variables were excluded from further analysis. A total of 290 out of 662 questionnaires were thus excluded, resulting in a sample size of 372 (questionnaire completion rate= 56.2% [372/662]).

### General Characteristics of the Respondents

Of the 372 respondents who had completed the e-questionnaire, 268 were female (72.0%), and 194 were in the age group of 18 and 30 (52.2%). Medical students made up about one-quarter of the sample (25.5%; 95/372), 15.9% (59/372) were nursing students, while 21.0% (78/372) were physicians and 37.6% (140/372) were nurses (Table 1).

A total of 181 out of 218 professionals (83.0%) and 115 out of 154 students (74.7%) were willing to use digital health tools for patient care. Among the professionals, almost 70.6% (154/218) had previously received training on using digital tools in clinical care, and about 62.8% (137/218) had prior experience of using digital tools on the departmental level (Mean years of experience = 13 years).

**Table 1:** Characteristics of the study population.

Characteristics		Health care professionals (N=218), n (%)	Health care students (N=154), n (%)		Total (N=372)
**Age (years**)					
	18-20	3 (1.4)		45 (29.2)	48 (12.9)
	21-25	6 (2.8)		107 (69.5)	113 (30.4)
	26-30	31 (14.2)		2 (1.3)	33 (8.9)
	31-35	61 (28.0)		0 (0.0)	61 (16.4)
	36-40	30 (13.8)		0 (0.0)	30 (8.1)
	41-45	37 (17.0)		0 (0.0)	37 (9.9)
	46-50	21 (9.6)		0 (0.0)	21 (5.6)
	Over 50	29 (13.3)		0 (0.0)	29 (7.8)
**Gender**					
	Male	53 (24.3)		51 (33.1)	104 (28.0)
	Female	165 (75.7)		103 (66.9)	268 (72.0)
**Willingness to use digital tools in patient care**					
	Yes	181 (83.0)		115 (74.7)	296 (79.6)
	No	37 (17.0)		39 (25.3)	76 (20.4)

### Perceived Benefits and Costs of Using Digital Health Tools Among Health Care Professionals and Students

Table 2 shows means of perceived benefits and perceived costs of using digital health tools in patient care among health care professionals and students. The most often perceived benefits by both, health care professionals and students, were increased quality of care, easy access to patient data, and increased work satisfaction. Regarding the perceived costs, health care professionals perceived that using eHealth tools would raise concerns about patient data safety, increase risk of technical errors, and increase financial costs for hospitals. Students, by contrast, perceived that use of eHealth tools would increase work-related stress, cause delay in the response to meet patients’ needs, and increase financial costs for hospitals.

Compared to students, health care professionals were more likely to perceive that using digital tools provides easier access to patient data (*P*=.01), higher number of patients turning up in time for their appointments (*P*=.03), and improvement in patients’ adherence to treatment (*P*=.009). Regarding potential costs health care professionals were more likely than students to perceive that using digital health tools in patient care would increase financial burden for hospitals (*P*=.03) as well as work-related stress among health care professionals (*P*=.01), and cause delay in the response of health care professions to meet patients’ needs (*P*=.02).

**Table 2:** Perceived benefits and costs of using digital health tools among health care professionals and students (N=372).

Perceived benefits and costs of using digital health tools			Health care professionals (N=218), mean (SD)		Health care students (N=154), mean (SD)		*t (df)*		*P* value
**Perceived benefits**									
	Quality of care	4.06 (0.9)		4.16 (0.9)		–0.93 (370)		.35	
	Easy access to patient data for health care professionals	4.16 (0.8)		3.92 (0.9)		2.60 (370)		.01	
	Work satisfaction among health care professionals	3.98 (1.0)		3.94 (0.9)		0.40 (370)		.68	
	Increased understanding of health conditions among patients	3.85 (1.0)		3.84 (1.0)		0.14 (370)		.88	
	Opportunities for self-care	3.64 (1.0)		3.69 (0.9)		–0.50 (370)		.61	
	Increased quality of communication between health care professionals and patients	3.83 (1.1)		3.60 (1.1)		1.84 (370)		.06	
	Higher number of patients turning up in time for their appointments	3.48 (1.2)		3.22 (1.1)		2.12 (370)		.03	
	Improved patients’ adherence to treatment	3.88 (0.9)		3.62 (0.9)		2.63 (370)		.009	
	Increased patient satisfaction	3.87 (0.9)		3.77 (0.9)		0.99 (370)		.32	
	Improved trust between health care professionals and patients	3.74 (1.2)		3.64 (1.1)		0.79 (370)		.42	
**Perceived costs**									
	Concerns about data safety among patients	3.76 (1.0)		3.62 (1.0)		1.26 (370)		.20	
	Increased risk of technical errors (eg, tool breakdown, internet breakdown)	3.53 (1.2)		3.67 (1.1)		–1.11 (370)		.26	
	Increased financial costs for hospitals	3.53 (1.3)		3.23 (1.3)		2.13 (370)		.03	
	Higher risk of data misuse	3.27 (1.2)		3.23 (1.1)		0.31 (370)		.75	
	Increased financial cost for patients	3.06 (1.2)		2.89 (1.1.)		1.58 (370)		.11	
	Higher risk of medical errors	2.94 (1.2)		2.79 (1.1)		1.17 (370)		.23	
	Increased level of anxiety among patients	2.81 (1.1)		2.66 (0.9)		1.34 (370)		.18	
	Increased demand of time for health care professionals	3.06 (1.2)		3.05 (1.1)		0.11 (370)		.90	
	Increased work-related stress among health care professionals	3.31 (1.2)		2.98 (1.1)		2.44 (370)		.01	
	Delay in the response from health care professions to meet patients’ needs	3.18 (1.2)		2.91 (1.0)		2.20 (370)		.02	

### Associations Between Willingness to Use Digital Health Tools in Patient Care and Sociodemographic Characteristics as well as Sociocognitive Factors

Tables 3 and 4 show the results of the bivariate analysis for willingness to use digital health tools and background characteristics as well as attitudes and beliefs among health care professionals and students, respectively.

Among health care professionals, being a nurse as opposed to being a physician was significantly associated with increased willingness to use digital tools in patient care (*P*=.03). Furthermore, significant positive associations with willingness to use digital tools were found for prior experience of using eHealth tools at the departmental level (*P*<.001), favorable attitudes (*P*<.001), perceived benefits (*P*<.001), and self-efficacy (*P*<.001) regarding personal use of these tools in patient care (Table 3).

Among students, being in the third or senior year as opposed to the second year was significantly associated with increased willingness to use digital tools in patient care (*P*=.01). Furthermore, significant positive associations with willingness to use digital tools were found for favorable attitudes (*P*=.01), perceived benefits (*P*<.001), and self-efficacy (*P*<.001) regarding personal use of these tools in patient care (Table 4).

**Table 3:** Bivariate associations between willingness to use eHealth tools and sociodemographic characteristics as well as sociocognitive factors among health care professionals.

Variables		Willing to use (N=181)	Not willing to use/uncertain about use (N=37)	*t* (*df*)	χ^2^ (*df*)	*P* value
**Age, n (%)**						
	Over 35 years	99 (54.7)	18 (48.6)	—^a^	0.45 (1)	.50
	35 years or below	82 (45.3)	19 (51.4)			
**Gender, n (%)**						
	Female	137 (75.7)	28 (75.7)	—	0.01 (1)	.99
	Male	44 (24.3)	9 (24.3)			
**Professional education, n (%)**						
	Nurses	122 (67.4)	18 (48.6)	—	4.70 (1)	.03
	Physicians	59 (32.6)	19 (51.4)			
**Ever received training on using digital tools in clinical care, n (%)**						
	Yes	130 (71.8)	24 (64.9)	—	0.71 (1)	.39
	No	51 (28.2)	13 (35.1)			
**Prior experience of using digital tools at the departmental level, n (%)**						
	Yes	124 (68.5)	13 (35.1)	—	14.65 (1)	<.001
	No	57 (31.5)	24 (64.9)			
Years of experience, mean (SD)		13.5 (8.7)	12.4 (8.1)	0.63 (172)	—	.52
Attitude toward using digital tools in patient care, mean (SD)		4.1 (0.7)	3.2 (0.9)	6.32 (216)	—	<.001
Perceived benefits of using digital tools in patient care, mean (SD)		3.9 (0.6)	3.2 (0.6)	5.70 (216)	—	<.001
Perceived costs of using digital tools in patient care, mean (SD)		3.2 (0.7)	3.2 (0.6)	–0.38 (216)	—	.70
Self-efficacy about personally using digital tools in patient care, mean (SD)		5.4 (1.3)	3.8 (1.5)	6.41 (216)	—	<.001

^a^—: Not available

**Table 4:** Bivariate associations between willingness to use eHealth tools and sociodemographic characteristics as well as sociocognitive factors among students.

Variables			Willing to use (N=115)		Not willing to use/uncertain about use (N=39)		*t (df)*		χ^2^ (*df*)		*P* value
**Age, n (%)**											
	Over 21 years	85 (73.9)		24 (61.5)		—^a^		2.15 (1)		.14	
	18-21 years	30 (26.1)		15 (38.5)							
**Gender, n (%)**											
	Female	75 (65.2)		28 (71.8)		—		0.56 (1)		.45	
	Male	40 (34.8)		11 (28.2)							
**Professional education, n (%)**											
	Nursing students	39 (33.9)		20 (51.3)		—		3.71 (1)		.053	
	Medical students	76 (66.1)		19 (48.7)							
**Current year of study, n (%)**											
	Third or senior years^b^	65 (65.0)		16 (41.0)		—		6.63 (1)		.01	
	Second year	35 (35.0)^a^		23 (59.0)							
Attitude toward using digital tools in patient care, mean (SD)			4.0 (0.8)		3.6 (0.9)		2.37 (152)		—		.01
Perceived benefits of using digital tools in patient care, mean (SD)			3.8 (0.6)		3.4 (0.7)		3.34 (152)		—		<.001
Perceived costs of using digital tools in patient care, mean (SD)			3.0 (0.6)		3.2 (0.5)		–1.40 (152)		—		.16
Self-efficacy about personally using digital tools in patient care, mean (SD)			5.1 (1.2)		4.02 (1.2)		4.16 (152)		—		<.001

^a^—: Not available

^b^N=100.

In the multivariable analysis for health care professionals, willingness to use digital tools in patient care was positively associated with attitude and self-efficacy. Among health care students, willingness to use digital tools in patient care was positively associated with self-efficacy and current year of study, with higher odds for third- or senior-year students compared to second-year students. No significant difference in willingness to use digital tools between nurses and physicians (*P*=.113), and between nursing and medical students (*P*=.079) was found. Furthermore, in both subsamples perceived benefits were no longer significant once attitudes were controlled for (Tables 5 and 6).

Another multivariable analysis conducted for the whole sample (ie, health care professionals plus students) found significant differences in willingness to use digital tools for perceived benefits (AOR 1.91; 95% CI 1.17-3.12) and self-efficacy (AOR 1.64; 95% CI 1.30-2.07). However, willingness to use digital tools did not vary significantly between the groups of health care professionals and students (*P*=.400; Multimedia Appendix 2).

**Table 5:** Multivariable analysis for health care professionals for the association between willingness to use eHealth tools and sociodemographic characteristics as well as sociocognitive factors.

Variables		Adjusted Odds Ratios (95% CI)	
**Age**			
	Over 35 years	1.82 (0.74-4.49)	
	35 years or below	Reference	
**Gender**			
	Female	0.71 (0.21-2.34)	
	Male	Reference	
**Professional education**			
	Nurses	2.35 (0.81-6.82)	
	Physicians	Reference	
**Prior experience of using digital tools at the departmental level**			
	Yes	2.40 (0.90-6.35)	
	No	Reference	
Attitude toward using digital tools in patient care		1.96 (1.14-3.36)	
Perceived benefits of using digital tools in patient care		1.90 (0.89-4.03)	
Self-efficacy about personally using digital tools in patient care		1.64 (1.17-2.30)	

**Table 6:** Multivariable analysis for health care students for the association between willingness to use eHealth tools and sociodemographic characteristics as well as sociocognitive factors.

Variables			Adjusted Odds Ratios (95% CI)
**Age**			
	Over 21 years	0.67 (0.21-2.12)	
	18-21 years	Reference	
**Gender**			
	Female	0.66 (0.25-1.75)	
	Male	Reference	
**Professional education**			
	Nursing students	0.43 (0.17-1.06)	
	Medical students	Reference	
**Current year of study**			
	Third or senior years	2.08 (1.18-3.68)	
	Second year	Reference	
Attitude toward using digital tools in patient care			0.69 (0.34-1.40)
Perceived benefits of using digital tools in patient care			2.20 (0.94-5.13)
Self-efficacy about personally using digital tools in patient care			1.77 (1.17-2.69)

## Discussion

### Principal Findings

Respondents had a largely positive view about the potential of eHealth tools for clinical practice. Interestingly, besides comparatively obvious advantages of eHealth tools, such as an easier access to patient data, it was most of all quality of care which was expected to benefit. Accordingly, large majorities in all subgroups expressed a willingness to use digital tools in patient care, with acceptance rates varying between 87.1% (122/140) and 75.6% (59/78) in nurses and physicians, and 66.1% (39/59) and 80.0% (76/95) in nursing and medical students, respectively. This is in line with findings from a previous study conducted in Saudi Arabia [35], which had more narrowly focused on telemedicine and reported that 78.9% of health care professionals were interested in adopting the technology for patient care [35]. Yet another Saudi Arabian study found an even higher telemedicine acceptance rate of 90.0% across several medical specialties, but comparability is limited because what was measured was not personal willingness but estimates of general acceptance/interest among physicians [34]. Apart from targeting a broader spectrum of eHealth applications beyond telemedicine, our study also enables a more differentiated view on subgroups of health care professionals in terms of professional background (medical doctors/nurses) and allows for comparisons between health care professionals and students.

In this study, multivariable comparisons between nurses and physicians showed no general differences in willingness to use eHealth tools. A few prior studies conducted in countries as different as Germany, Lebanon, and the United States have reported that physicians are more likely to use or accept eHealth tools compared to other health care professionals [12,19,37]. However, these studies were focused on specific tools, such as telemedicine [12] or use of eHealth records [37] or were conducted in settings very different from the present one (ie, primary health care) [19] or in very specific medical areas, such as pregnancy monitoring [12,37], which limits comparability. By contrast, another study, which also investigated willingness to engage with eHealth in general, did not find differences between professional groups, either [17]. In addition, it needs to be noted that our study was conducted at one of the largest and technologically most advanced university hospitals in the country which might have involved a more homogeneously motivated and “open” group of health care professionals compared to other, more diverse settings. Nurses in this hospital are already familiar with using digital tools for administrative purposes.

Similarly, we did not find an association between extent of professional experience (number of years in the profession) and willingness to use eHealth. By contrast, the previous study by El-Mahalli et al [35], which investigated not only willingness but also actual adoption of telemedicine by health care professionals in Saudi Arabian hospitals, suggested that compared to more junior physicians and nonphysicians, senior consultants with more than 20 years’ experience were significantly more likely to use telemedicine. This discrepancy between the 2 studies is most likely explained by their different focus on telemedicine versus eHealth in general. Willingness to practice telemedicine might require more long-standing professional experience with patients and resultant self-confidence regarding diagnosis/treatment than use of other eHealth tools, such as electronic patient records, webinars, or monitoring apps. Besides, the acceptance and use of telemedicine as well as other digital tools might have increased significantly over the years among health care professionals of all age group and years of experience [38].

Further, previous use of eHealth tools at the departmental level, which can be seen as a proxy variable for personal experience, was not identified as a relevant factor in this study. The initial bivariate test had indicated a significant association (*P*<.001), which however was not confirmed in the multivariable analysis. It is likely that the effect of prior experience was mediated by attitudes as well as self-efficacy, which might have increased with experience, so that after adjustment for these factors in the multivariable analysis, prior experience was not significant anymore.

Among the subgroup of students, willingness to use digital tools did not significantly differ in terms of sociodemographic characteristics such as age, gender, and professional education (medicine versus nursing). However, willingness was positively associated with current year of study, with higher odds for third- or senior-year students compared to second-year students. At KSU, the subject “medical informatics” is taught in the third year of study for medical students, and recently, a new subject “nursing informatics” has been offered to third-year nursing students. Thus, the association between year of study and increased willingness to use eHealth tools emphasizes the importance of a structured, formal eHealth curriculum for health care students [39]. Noor [40], based on the data from a survey study, reported that only 10 out of 109 higher education institutions in Saudi Arabia provide specific courses on medical informatics. Further, the author noted a need for standardized, accredited programs in this field as well as integrating well-structured eHealth courses into nursing and medical education programs and health care practice.

As for the role of expected benefits and positive attitudes, the findings of our study are in line with past research [16,21] and theoretical models, such as the Technology Acceptance Model [41] or the related Unified Theory of Acceptance and Use of Technology [42], which have specifically emphasized the role of “perceived usefulness of technology for intention to use and actual use.” However, this study also indicates some specificity of effects for different subgroups. Thus, positive attitudes in terms of the importance attributed to different eHealth tools for patient (self-) management were related to higher willingness to use these tools among professionals but not the students. This is plausible insofar students, because of their lack of clinical practice and experience, might be more uncertain than health care professionals about the importance of eHealth tools for patients. These observations were also made by Wernhart et al [11] in their study investigating differences in perceptions regarding eHealth and telemedicine among health care professionals and students at the teaching hospital of the University of Vienna, Austria.

Regarding the motivation of health care professionals to use eHealth tools, it is further interesting to note that the tools’ potential to actually benefit patient (self-) management makes a difference, while the sum of different expected positive consequences for the hospital, doctors/nurses, or patients were less relevant in comparison. This factor had been significant (*P*<.001) in the bivariate analysis and showed a very similar trend for an effect like attitudes in the multivariable analysis, but lost significance once attitudes were controlled for. For one thing, due to their clinical experience, health care professionals might feel more certain about specific effects of eHealth for patients than for the hospital or work conditions in general [25]. Additionally, physicians’ and nurses’ professional role identity as patient advocates might make the function of eHealth tools to serve patients’ needs particularly salient. Besides, it might be argued that an implementation of eHealth tools via improving patients’ self-management capacities will also facilitate physicians’ and nurses’ work.

Interestingly, an additional analysis combining the health care professionals with the students showed a somewhat different result. The similar nonsignificant trends for perceived benefits in both subgroups added up to a significant effect based on the higher statistical power due to increased sample size, while the positive effect for attitudes, which was only present in health care professionals, was no longer relevant. This indicates the importance of subgroup-specific analysis, because actual differences might otherwise be missed.

Of note, perceived costs/negative consequences did not seem to make a difference. This finding does, however, not imply that perceived costs might be similarly irrelevant when it comes to actual adoption [13,25]. General willingness might mainly require a positive motivation or a clear rationale in favor of change while costs might become more apparent once actual experience is initiated.

Finally, we found that evaluation of own competences to use eHealth tools is important for their adoption by health care professionals as well as by students. The relevance of self-efficacy has also been shown by previous studies [7,16,28]. This emphasizes the need for capacity building in eHealth for both professionals and students, which can be achieved by regular education, training, and evaluation with feedback [7]. This would, however, require support structures, which in a recent review were identified as still lacking in most Middle Eastern countries, including Saudi Arabia [43].

Although implementation of some types of eHealth tools, such as electronic medical records and telemedicine, has been discussed or promoted on a political level, such tools have not yet been adopted in Saudi Arabia on a large scale [33-35]. The findings of this study suggest that most nurses, physicians as well as medical and nursing students are generally willing to adopt eHealth tools in patient care, which is an important prerequisite for the successful implementation of eHealth interventions. Further, education programs about eHealth interventions and their potential positive consequences for patients as well as programs teaching skills to competently use eHealth tools may enhance current and future health care professionals’ readiness to adopt such interventions. The present findings, which indicated no systematic differences between males/females, age groups, or physicians and nurses, also suggest that eHealth educational and promotional activities should take a broad, inclusive approach, targeting health care professionals in general, irrespective of their gender and professional background.

### Limitations

This study has some limitations. First, because of the cross-sectional nature of the study we were not able to establish causality beyond plausibility assumptions. Further, data were collected via an e-survey method with a response rate of 22.02% (662/3007) and a questionnaire completion rate of 56.2% (372/662), which limits the generalizability of the study results. This response rate is lower than the rates reported by the previous Saudi Arabian studies by Albarrak et al [34] and El-Mahalli et al [35]. This might be due to the considerable length of the questionnaire, which included not only standard single items but also several multi-item instruments to enable a valid assessment of sociocognitive factors. The overall low response suggests that the high level of willingness to use e-tools may to some extent be due to an overrepresentation of those who already held more positive attitudes about and a stronger interest in eHealth and were therefore more motivated to participate in the first place or to complete the questionnaire in full.

In addition, although all respondents were assured about anonymity/confidentiality, we cannot be certain that responses were not influenced by social desirability. Because respondents probably were aware that eHealth was considered a desirable strategy by the hospital leadership, they might have responded more positively. Two of the response categories for the outcome “willingness to use” had to be merged, that is “not sure” responses were collapsed with the “no” responses. Therefore, the results can only be interpreted as “full willingness” versus “uncertain about use plus nonwillingness.” Finally, we were not able to take into account the potential impact of local contextual factors on health care professionals’ decisions about using digital tools. Investigating such context factors (eg, cultural factors, social influences), as well as the needs and the level of eHealth literacy of the patient groups was out of the scope of this study and should be addressed by future research.

### Conclusions

Our findings suggest that most nurses, physicians as well as medical and nursing students in a major Saudi Arabian university hospital are willing to adopt eHealth tools for patient care. The most important factors with respect to motivation for adoption by health care professionals are favorable attitudes related to positive impacts for patients and a sense of self-efficacy. For those still undergoing education, being a senior student and having self-efficacy are most relevant. The findings of this study should encourage policy makers and hospital managers in Saudi Arabia to introduce and implement relevant eHealth interventions into routine health care programs. In addition, students as well as current health care professionals should be targeted by eHealth education programs with an aim to develop required positive attitudes. In particular, the aim is to create awareness about the value that eHealth tools can have for many patients as well as to promote and refine practical skills. Future research should include organization-level factors which might facilitate or hinder eHealth implementation as well as willingness among patients to use these tools.

## Appendix

Multimedia Appendix 1 Characteristics of nonresponders.

Multimedia Appendix 2 Multivariable models for the association between willingness to use eHealth tools and sociodemographic characteristics and sociocognitive factors (both health care professionals’ and students’ groups combined).

## Abbreviations

AOR: Adjusted Odds Ratios
KSU: King Saud University
KSUMC: King Saud University Medical City Hospital
WHO: The World Health Organization
