# Supplementary material for: Willingness to Use Digital Health Tools in Patient Care Among Health Care Professionals and Students at a University Hospital in Saudi Arabia: Quantitative Cross-sectional Survey
Source: JMIR Med Educ. 2021 Feb 19;7(1):e18590. doi: 10.2196/18590 (PMC8081256; doi:10.2196/18590)
Supplement: Multimedia Appendix 1 [file mededu_v7i1e18590_app1.docx]

**Multimedia Appendix 1.** Characteristics of nonresponders.

|  | Responders  (n=372)  n (%) | Non-responders  (n=290)  n (%) | *χ^2^*  *P* value |
| --- | --- | --- | --- |
| **Gender** |  |  |  |
| Female | 268 (72.0) | 187 (64.5) | <.001 |
| Male | 104 (28.0) | 103 (35.5) |  |
| **Age** |  |  |  |
| 30 or below | 194 (52.1) | 199 (68.6) | <.001 |
| Over 30 | 178 (47.8) | 91 (31.4) |  |
| **Background** |  |  |  |
| Professionals | 218 (58.6) | 119 (41.0) | <.001 |
| Students | 154 (41.4) | 171 (59.0) |  |
