# Supplementary material for: Willingness to Use Digital Health Tools in Patient Care Among Health Care Professionals and Students at a University Hospital in Saudi Arabia: Quantitative Cross-sectional Survey
Source: JMIR Med Educ. 2021 Feb 19;7(1):e18590. doi: 10.2196/18590 (PMC8081256; doi:10.2196/18590)
Supplement: Multimedia Appendix 2 [file mededu_v7i1e18590_app2.docx]

**Multimedia Appendix 2.** Multivariable models for the association between willingness to use eHealth tools and sociodemographic characteristics and sociocognitive factors (both health care professionals’ and students’ groups combined).

| Variables | AOR (95% CI) ^i^ |
| --- | --- |
| Age (over 30/30 or below) | 1.05 (0.38 – 2.87) |
| Gender (female/male) | 1.02 (0.38 – 1.94) |
| Professional background (health care professionals/ students) | 1.52 (0.57 – 4.08) |
| Attitude towards using digital tools in patient care | 1.24 (0.86 – 1.80) |
| Perceived benefits of using digital tools in patient care | 1.91 (1.17 – 3.12) |
| Self-efficacy about personally using digital tools in patient care | 1.64 (1.30 – 2.07) |

AOR: Adjusted Odds Ratio; CI: Confidence Interval
